# Multifactorial Effects of Gelling Conditions on Mechanical Properties of Skin-Like Gelatin Membranes Intended for In Vitro Experimentation and Artificial Skin Models

**DOI:** 10.3390/polym13121991

**Published:** 2021-06-18

**Authors:** Lilian C. Alarcón-Segovia, Jorge I. Daza-Agudelo, Ignacio Rintoul

**Affiliations:** 1 Instituto de Desarrollo Tecnológico para la Industria Química, Universidad Nacional del Litoral and Consejo Nacional de Investigaciones Científicas y Técnicas, Santa Fe 3000, Argentina; sterbenkaiser@gmail.com; 2 Núcleo de Innovación Médica, Facultad de Medicina, Universidad María Auxiliadora, Asunción 2040, Paraguay

**Keywords:** artificial skin models, animal alternative, animal-free testing, skin equivalent

## Abstract

The development of new cosmetic products, skin contact medical devices, skin medicaments, wound care devices, tattooing and piercing has experienced an impressive growth in recent years. In parallel, new restrictions to in vivo experimentation in animals and humans have been widely implemented by regulatory authorities. New knowledge about alternative materials for in vitro skin-related experimentation is required to overcome these severe limitations. This paper presents a set of three 4-D surface response equations describing the mechanical properties of skin-like gelatin membranes intended for use as an alternative biomaterial for in vitro skin-related experimentation. The membranes were obtained by a sol-gel method. The novelty of this contribution is the establishment of the cross-dependency effects of key synthesis conditions on the final mechanical properties of gelatin membranes. The results of this work are useful to produce gelatin membranes with tailored mechanical properties mimicking different types of human skins. In particular, membranes with Young’s modulus of 1 MPa and maximum tensile strength of 0.85 MPa were obtained.

## 1. Introduction

The skin tissue is the largest organ of the human body. It represents about 15% of the body weight of an adult person. Skin is a membrane gland and performs absorption, secretion, respiration, temperature regulation, general and special sensitivity and synthesis of vitamin D, among many other functions [1,2,3].

Skin is composed of two layers. The outer layer is the epidermis and the inner layer is the dermis [4,5,6]. The thickness of the epidermis is between 0.05 mm and 1 mm depending on sex, age and anatomical location. The epidermis is itself composed of several sub-layers. The basal layer, the spinous layer, the granular layer, the lucid layer and the horny layer [7,8,9,10]. The dermis is four to five times thicker than the epidermis, provides the supporting tissue of the skin and determines the mechanical properties of the skin tissue [8].

A key mechanical property is the Young’s Modulus (YM). The average YM of skin is 1 MPa [2,3,6]. Maximum tensile stress (MTS) and rupture ultimate strain (RUS) are also of utmost importance and their values greatly depend on the anatomical location [11,12,13,14]. Intensive knowledge about skin mechanical properties is very important to develop new cosmetic products, devices using microneedles and microjets and for the diagnosis of various skin diseases [9,10,15].

The development of clinical and cosmetic products and skin-related medical devices requires in vivo, ex vivo and in vitro experimentation. In vivo evaluation involves experimentation in humans and animals. These experiments are very expensive, require many test repetitions and ethical permissions. Ex vivo experiments use human skin tissues from cadaver and surgical sources or skin tissues from vivarium porcine, rabbit or rat sources. In vitro experiments use artificial skin models, such as liquid suspensions, gelatinous substances, elastomers, epoxy resins and textiles, with human-skin-mimicking properties [16,17,18,19].

Gelatin is obtained by the hydrolysis denaturation of animal connective tissues, such as skin, bone and tendons [20,21]. Gelatin is biocompatible, cheap and is used in various applications in the food, pharmaceutical and medical industries [22,23,24]. Gelatin has an excellent membrane-forming capacity. A recent sol-gel method to obtain gelatinous membranes has been reported in the literature [25,26]. The toughness of gelatin membranes can be increased by introducing small plasticizing molecules. The function of plasticizers is to reduce the intermolecular interactions between the gelatin chains, increasing their flexibility, workability and dispensability. It also usually reduces the deformation strain, hardness, density, viscosity and electrostatic charge of the polymer, while increasing the flexibility of polymer chains, fracture toughness and dielectric [27,28]. Glycerol is a commonly used plasticizer due to its high efficiency, rapid diffusion and interaction with gelatin molecules. Other commonly used plasticizers are lipids and water [29].

Several in vitro studies have reported the use of water-interacting substances with the ability to form gels with physical properties comparable to those of human tissues [17,18]. For instance, gelatin membranes have been used in ophthalmic treatments [30] and for controlled skin release of ciprofloxacin [31]. Moreover, ballistic gelatins have been used as customizable artificial tissue models [19,32,33]. These studies reported the change of several mechanical and electrical properties according to the synthesis parameters, including the relative amounts of water, gelatin and salt used to compose the gel material. Other studies reported the preparation of a gelatin-based substrate with surface chemistry, roughness, wettability and hydration similar to that of the human skin [34]. Another artificial tissue model has been developed by mixing different amounts of gelatin, glycerol, polysaccharides and lipids. The resulting gels were able to mimic the mechanical and surface properties of human skin and were used to study the adhesion of different substrates to skin [35]. It is clear from the mentioned references that synthesis parameters have cross-dependency effects on the final properties of the resulting materials. Therefore, the usual way of experimentation varying one-parameter-at-a-time is very limited for the full comprehension of the correlation between synthesis conditions and resulting mechanical properties of gelatin membranes. 

This work presents a comprehensive model of the critical parameters affecting the mechanical properties of skin-like gelatin membranes (SLGMs). The ultimate goals are the correlation of the synthesis parameters of SLGMs with their resulting mechanical properties and the finding of recipes resulting in SLGMs with mechanical properties similar to those of human skin. The obtained SLGMs are designed to serve as a model for in vitro skin experimentation. The model uses key parameters controlling the synthesis of the gelatin membranes as input data and defines the YM, MTS and RUS as output results. The principal results of the paper is a set of equations expressing the mechanical properties of SLGMs (YM, MTS and RUS) as functions of their synthesis conditions.

## 2. Materials and Methods

### 2.1. Materials

Technical-grade type I B gelatin (alkaline conditioned gelatin derived from bovine leather) with Bloom 180-260 (AN-MAX FG3 PB Liner, Santa Fe, Argentina), glycerol 87% pro-analysis (Cicarelli, San Lorenzo, Argentina) and MilliQ water with resistivity of 18.2 MΩ and density δw = 0.99704 g cm^−3^ were used as forming polymer, plasticizer and solvent, respectively.

### 2.2. Synthesis of SLGMs

SLGMs were prepared by a modified sol-gel method [36,37]. First, the forming polymer was dissolved in MilliQ water. Second, the solution was stirred at room temperature. Third, a certain amount of plasticizer was added to the solution. Then, the solution was poured on a polycarbonate Petri dish and placed in a stove conditioned at 40 °C and 50% of relative humidity until constant weight. Finally, the formed SLGMs were removed from the dishes and stored in a hermetically sealed container. The gelatin content, the glycerol content, the time of maturation and the amount of water were selected as synthesis variables.

### 2.3. Sol-Gel Method Conditions

Table 1 presents the synthesis conditions applied to the sol-gel method according to a Taguchi L9 experimental design.

### 2.4. Thickness of SLGMs

The thickness of SLGMs was measured using a Precision Micrometer (Testing Machines Inc., New Castle, USA). Each sample was composed of 10 membranes individually obtained. The membrane thickness was measured in five random locations of each membrane. The average thickness determination demanded 50 thickness measurements. The thickness values are needed to determine the mechanical properties of SLGMs.

### 2.5. Mechanical Properties

The mechanical properties of the SLGMs were measured using a universal mechanical testing machine (INSTRON 3344 Q 1469, Norwood, MA, USA) equipped with a 100 N load cell applying the ASTM D882-12 norm. The tests were performed in a laboratory conditioned at 23 °C and 50% of relative humidity. The specimens were withdrawn from the SLGMs by punching normalized double T-shaped samples. The width and the length of the samples were 7 mm and 22 mm, respectively. The specimens were stretched to the breaking point at an elongation rate of 10 mm h^−1^. The engineering stress in the specimens was calculated as the stretching force measured by the machine divided by the force’s perpendicular initial area of the specimen. The force’s perpendicular initial areas were calculated as the width of the specimens multiplied by their corresponding thickness. The YM of SLGMs were calculated from the slope of the initial linear section of the stress–strain plots. The MTS and RUS were calculated using Equations (1) and (2), respectively.

(1) $$\mathrm{MTS}=\mathrm{RUS}\times {\left(\mathrm{CS}\right)}^{-1}$$

(2) $$\;\mathrm{US}=\left(l_{f}-l_{0}\right)\times l_{0}^{-1}$$

Here, CS, *l_0_* and *l_f_* are the cross-section area, the initial length and the length at failure of the specimens.

### 2.6. ATR-FTIR Spectroscopy

The interaction between functional groups of the forming polymer and the plasticizer were analyzed by attenuated total reflectance Fourier transformed infra-red spectroscopy (ATR-FTIR) using a Fourier Transform Infra-red Spectroscope (FTIR-8201 PC-Shimadzu, Tokio, Japan).

### 2.7. Thermal Analysis

The thermal analysis of the SLGMs was performed using a thermobalance (Q500 TA Instruments, New Castle, DE, USA). The samples were analyzed in the temperature range between 25 °C and 600 °C, with a heating rate of 10 °C min^−1^ under a nitrogen atmosphere (90 mL min^−1^).

### 2.8. Theoretical Calculations

The experimental conditions were selected using the Taguchi L9 method [38]. This method is usually used to study processes involving a considerable number of simultaneously changing variables. The method proposes that the responses resulting from the process (Y) can be expressed as polynomial functions of its variables [35,36]. The responses can be expressed according to Equation (3).

(3) $${Y=\Sigma }_{h,k,l,m}a_{h,k,l,m}\cdot A^{h}\times B^{k}\times C^{l}\times D^{m}$$

Each term of Equation (6) is composed of the product of an empirical coefficient (*a*_h,k,l,m_), determined by a least squares adjustment and the process variables (*A*, *B*, *C* and *D*) raised to their corresponding non-negative integer exponents (*h*, *k*, *l* and *m*). The greater the complexity of the response to a given stimulus, the greater the exponents of the terms and the greater the number of terms in the summation. The resulting polynomial function can have infinite terms and eventually describe any Y(A, B, C, D) functionality. 

In this work, the following process parameters have been defined. A: gelatin content (g), B: water content (mL), C: glycerol content (mL) and D: maturation time (min). Each variable was set to three different levels. Therefore, the number of experiments was limited to nine. Nine experiments give the possibility of defining a polynomial function of nine terms. The selection of terms was carried out using experimental raw data and iterated with Minitab 17 software. Minitab 17 software is mathematical software that solves equation systems easily. The YM, MTS and RUS responses were studied and described in 4-D surfaces defined in the space of the mentioned variables. Subsequently, 2-D plots were used to conclude and predict properties of resulting SLGMs to the simultaneous variation of their reaction conditions.

### 2.9. Surface Morphology

The morphologies of the surfaces of the obtained SLGMs were observed using a scanning electron microscope (JEOL JSM-35C, Tokio, Japan) equipped with powerful image software (JEOL Sem Afore, Tokio,, Japan).

## 3. Results and Discussion 

### 3.1. Mechanical Characterization of SLGMs

Table 2 shows the thickness, YM, MTS and RUS of SLGMs samples S1 to S9. 

The differential synthesis conditions allowed us to obtain membranes with YM between 0.06 MPa and 1.18 MPa. The membranes presenting YM in the ranges of 0.50–1.00 MPa, 0.20–0.50 MPa and 0.05–0.20 MPa can be used as mechanical models for artificial skin, muscles and nervous tissues, respectively [14]. According to this classification, materials obtained with the formulas S6, S8 and S9 can be used as mechanical models for skin studies; materials obtained with the formulas S1, S4, S5 and S7 can be used as mechanical models for muscles and the materials obtained with the formulas S2 and S3 can be used as mechanical models for nervous tissues. All membranes showed elastic behavior with elongation at rupture between 33.6% and 225.9%.

### 3.2. Chemical Characterization of SLGMs

**Figure 1:**
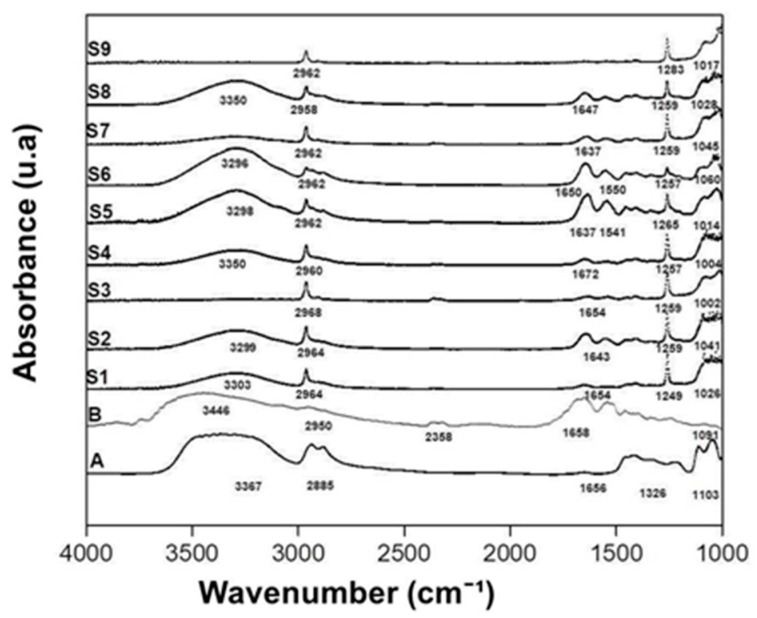
ATR-FTIR for: A: gelatin, B: glycerol and samples S1 to S9.

Figure 1 and Table 3 show the infra-red spectra and the identification of peaks and functional groups assignment of pure gelatin, pure glycerol and samples S1 to S9. 

The occurrence of a peak at 1249–1283 cm^−1^ in samples S1 to S9 is clear. Such peaks can be associated with the formation of tropocollagenic bindings between gelatin molecules. This result is proof of the formation of a gel structure. Samples S1, S2, S4, S5, S6, S7 and S8 show signs of hydroxyl groups, amide I, amide II and amide III located in the regions 3000–3500 cm^−1^, 1640–1650 cm^−1^, 1539–1550 cm^−1^ and 1033–1065 cm^−1^, respectively. The peaks positions and intensities are well-correlated with the interaction between polyols and proteins reported in the literature [39,40,41]. Sample S9 presents a displacement in the region of the hydroxyl group and sample S3 presents a displacement in the region of amide I and amide II. These displacements can be attributed to the fact that the time of formation of these membranes may not have been sufficient to establish an interaction between the polyols and the gelatin protein segments.

### 3.3. Thermal Analysis of SLGMs

**Figure 2:**
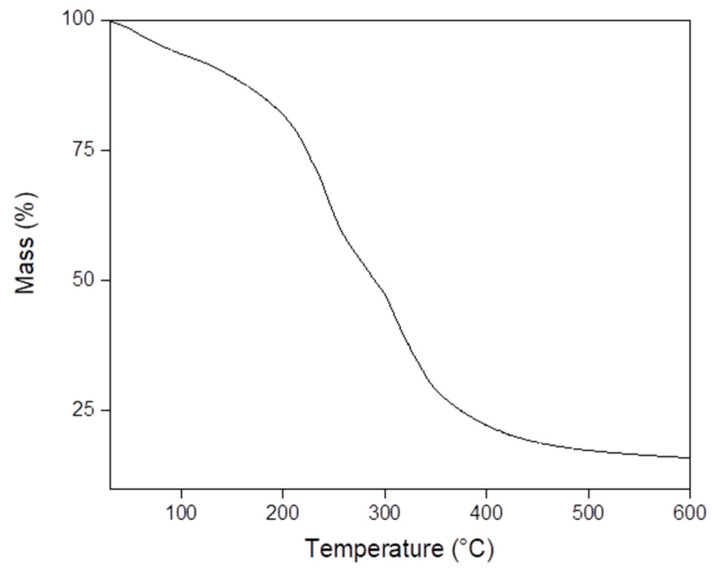
TGA analysis of sample S6.

**Figure 3:**
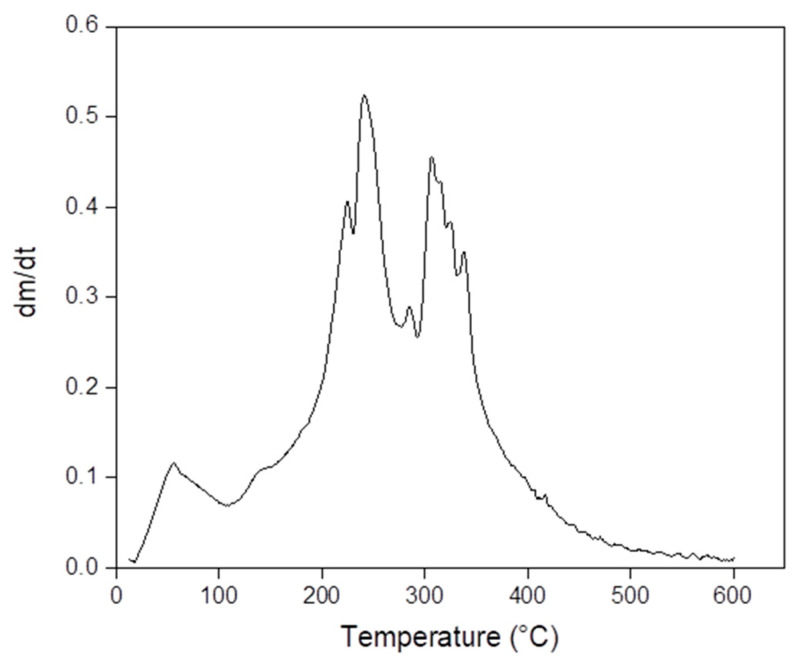
DTG analysis of sample S6.

S6, S8 and S9 were selected for thermal analysis because they presented YM in the range suitable to be used as a mechanical model for artificial skin. Exemplarily, Figure 2 and Figure 3 present TGA and DTG analysis of S6, respectively. TGA results showed 6%, 6% and 5% initial weight loss at 100 °C due to the evaporation of free water in samples S6, S8 and S9, respectively. Moreover, results also showed 23%, 12% and 28% of weight loss at 225 °C due to the evaporation of glycerol in samples S6, S8 and S9, respectively. Interestingly, sample S8, which has the highest YM, presented the lowest weight loss at 225 °C. This observation may indicate that the S8 formula promoted the highest degree of glycerol bridging of gelatin chains. Then, glycerol molecules incorporated into the gel structure are not able to evaporate at 225 °C. Finally, the decomposition of gelatin occurred between 290 °C and 450 °C for all samples. The DTG plot established the decomposition temperature at 241 °C [42,43].

### 3.4. Response Surface Analysis

The coefficients of the 4-D surface responses of the YM, MTS and RUS to the stimuli of the simultaneous variation of the gelatin content (A), water content (B), plasticizer content (C) and maturation time (D) are presented in Table 4. 

### 3.5. Young’s Modulus

**Figure 4:**
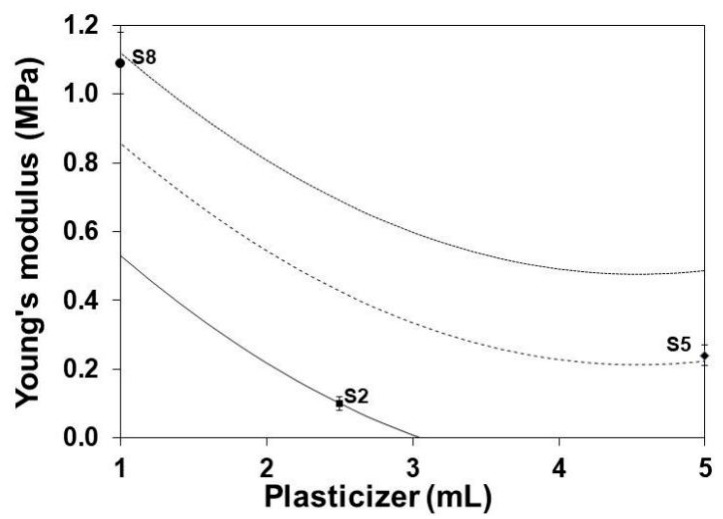
Effect of plasticizer on the YM of SLGMs varying the gelatin content: 1.0 g (continuous), 2.5 g (dashed) and 5.0 g (dotted). Experimental points: S2, S5 and S8. Conditions: water content = 50 mL; maturation time = 7.5 min.

**Figure 5:**
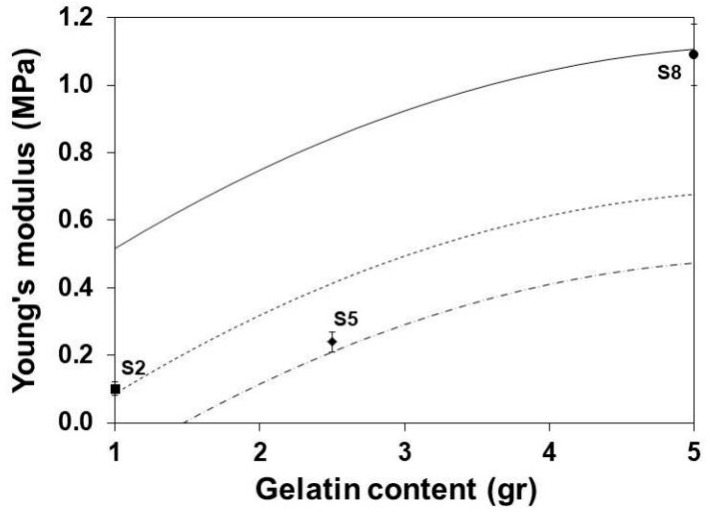
Effect of gelatin content on the YM of SLGMs, varying the glycerol content: 1.0 mL (continuous), 2.5 mL (dashed) and 5.0 mL (dotted). Experimental points: S2, S5 and S8. Conditions: water content = 50 mL; maturation time = 7.5 min.

Figure 4 and Figure 5 show the reduction of the YM with the increase of the glycerol content and the decrease of the gelatin content, respectively. The functions of gelatin as structure polymer [27] and the glycerol as plasticizer [44] are evident. The observed effects are associated with the ability of glycerol to form hydrogen bridges [45,46,47,48] and to reduce intermolecular forces with the protein segments of gelatin molecules [49,50].

### 3.6. Maximum Tensile Stress

**Figure 6:**
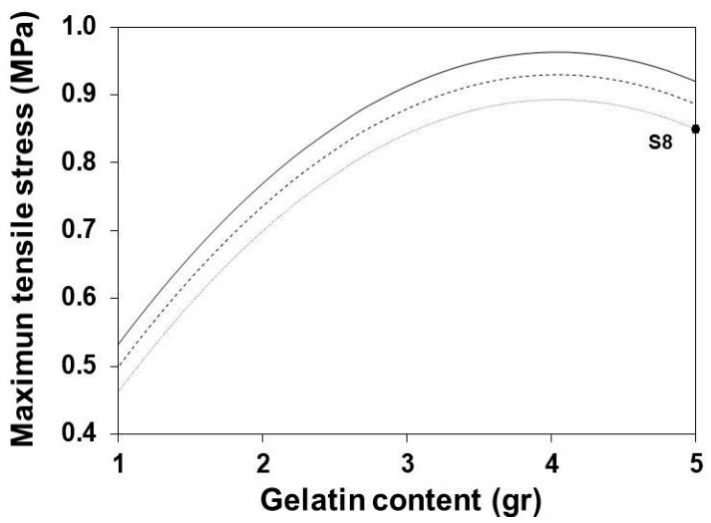
Effect of gelatin on the MTS of SLGMs, varying formation time 1.0 min (continuous), 7.5 min (dashed) and 15.0 min (dotted). Experimental point: S8. Conditions: water content = 50 mL; plasticizer content = 1.0 mL.

Figure 6 shows the variation of the MTS with the increase of the gelatin content at different maturation times. The increase of gelatin content increases the value of the MTS until it reaches a maximum gelatin content of ~3.9 g. In this case, the excess gelatin content may impair its homogeneous mixture, with the limited amount of glycerol generating a lack of internal cohesion. The increment of maturation times reduces the MTS. This effect can be attributed to some extent of hydrolysis of the protein chains in the gelatin structure [51,52,53].

**Figure 7:**
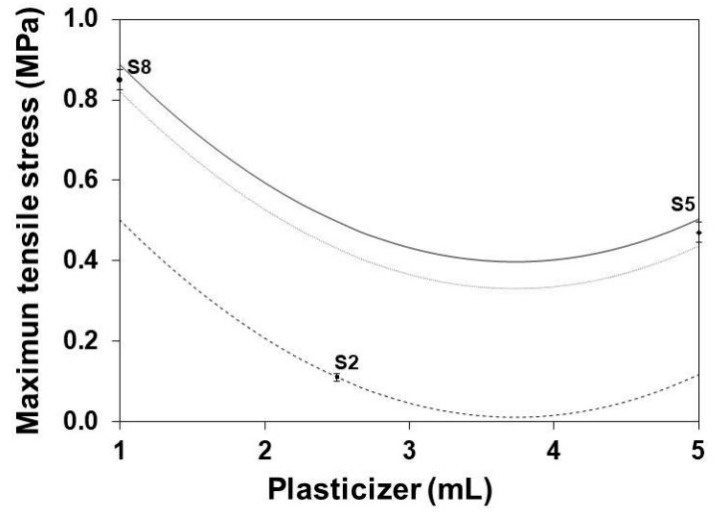
Effect of plasticizer on the MTS of SLGMs, varying the gelatin content: 1.0 g (continuous), 2.5 g (dashed) and 5.0 g (dotted). Experimental points (S2, S5 and S8). Conditions: water content = 50 mL; plasticizer content = 1.0 mL.

Figure 7 shows the influence of the glycerol content on the MTS of SLGMs obtained at three different gelatin contents. The MTS decreases with the increase of the glycerol content until it reaches a minimum at a glycerol content of ~3.8 mL. This behavior can be attributed to the initial plasticizer-dominant function of glycerol at low glycerol content followed by the increase of the hydrogen-bridging function at high glycerol contents [36].

### 3.7. Rupture Ultimate Strain

**Figure 8:**
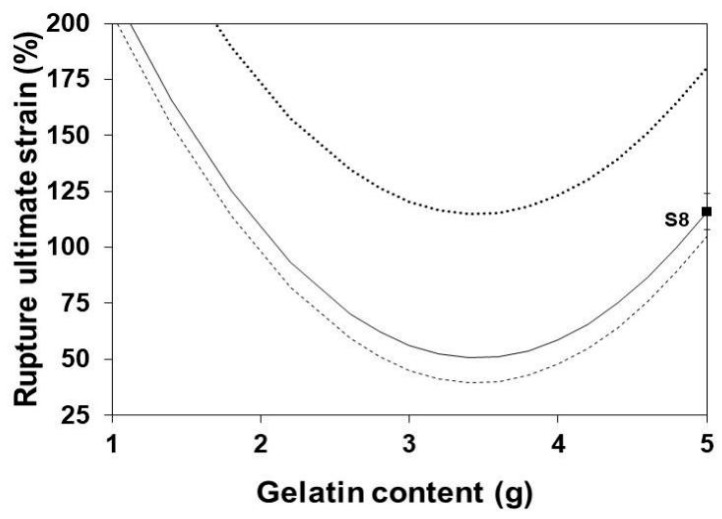
Effect of gelatin content on the RUS of SLGMs, varying the plasticizer content: 1.0 mL (continuous), 2.5 mL (dashed) and 5.0 mL (dotted). Experimental point: S8. Conditions: water content = 50 mL; maturation time = 7.5 min.

Figure 8 shows the influence of the gelatin content on RUS at three levels of glycerol content. Clearly, the RUS of SLGMs increases with the increment of the glycerol content. However, such increment seems to reach an upper plateau. The effect can be attributed to the saturation, by an excess of gelatin, of the possible sites for intermolecular bonding between protein segments of the gelatin structure. The increase of glycerol decreases the RUS to an apparently low plateau. In this case, further increment of glycerol do not alter the already established bonding between protein segments.

**Figure 9:**
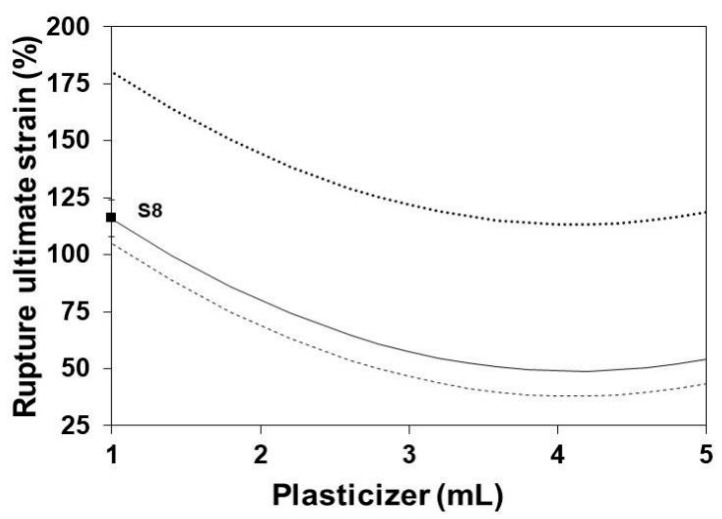
Effect of plasticizer on the RUS of SLGMs, varying the maturation time: 1.0 min (continuous), 7.5 min (dashed) and 15.0 min (dotted). Experimental point: S8. Conditions: water content = 50 mL; gelatin content: 5.0 g.

Figure 9 shows the influence of the glycerol content on the RUS of SLGMs at three maturation times. RUS slightly decreases with the initial increase of the glycerol content. This is due to the increasing separation of the protein segments as a consequence of the insertion of increasing amounts of glycerol within the gelatin structure. This situation causes a decrease in the cohesion of the gelatin structure, which in turn is evidenced as a decrease of the RUS. The increase of maturation time enhances the decrease of RUS by adding some extension of hydrolysis among the protein segments of the gelatin structure [50].

### 3.8. Surface Morphology

**Figure 10:**
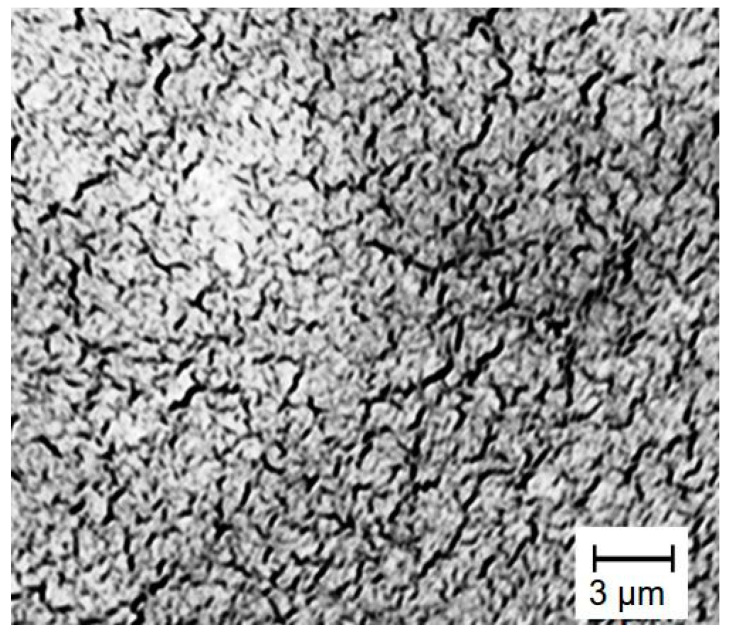
SEM image of sample S6.

The morphology of the surface of the membranes was observed in SEM images. No substantial differences in morphology were observed in samples S1 to S9. The preparation method seems not to have an important effect on the surface morphology of SLGMs. Figure 10 exemplary shows the surface morphology of sample S6. Interestingly, a superficial morphology similar to that of human skin is observed. The surface of the SLGMs presents a quite regular pattern of grooves homogeneously distributed over an apparently smooth surface. This observation finds its analog in human skin, where grooves, similar in dimensions to those in SLGMs, are also present in regular patterns. 

## 4. Conclusions

The modified sol-gel method presented in this contribution is useful to obtain SLGMs with mechanical properties according to those required for in vitro studies and applications. The cross-dependency of synthesis parameters in the resulting mechanical properties of obtained membranes was investigated. A set of equations in the form of 4-D surfaces was developed. The surface equations relate the gelatin, water and glycerol contents and the maturation time set during the preparation of the SLGMs to their resulting YM, MTS and RUS. The results were discussed in terms of the effects of gelatin, water, glycerol and maturation times in the formation of the molecular structure of SLGMs. The 4-D surface response set of equations permits to predict precisely the YM, MTS and RUS of resulting SLGMs and may be useful to produce tailored SLGMs for in vitro skin-related tests and experimentation.

## Figures and Tables

**Table 1:** Experimental design.

Sample	Parameters			
	A (g)	B (mL)	C (mL)	D (min)
S1	1.0	25	1.0	1.0
S2	1.0	50	2.5	7.5
S3	1.0	75	5.0	15.0
S4	2.5	25	2.5	1.0
S5	2.5	50	5.0	7.5
S6	2.5	75	1.0	15.0
S7	5.0	25	5.0	1.0
S8	5.0	50	1.0	7.5
S9	5.0	75	2.5	15.0

A: gelatin content, B: water content, C: glycerol content and D: maturation time.

**Table 2:** Mechanical properties of SLGMs obtained using different synthesis conditions.

Sample	Thickness (mm)	YM (MPa)	MTS (MPa)	RUS (%)
S1	0.40 ± 0.03	0.32 ± 0.03	0.30 ± 0.02	0.97 ± 0.13
S2	0.67 ± 0.05	0.10 ± 0.02	0.11 ± 0.01	0.34 ± 0.01
S3	0.58 ± 0.02	0.08 ± 0.01	0.03 ± 0.01	0.34 ± 0.06
S4	0.69 ± 0.01	0.26 ± 0.04	0.17 ± 0.01	0.88 ± 0.18
S5	0.96 ± 0.05	0.24 ± 0.03	0.47 ± 0.03	1.34 ± 0.49
S6	0.40 ± 0.02	0.90 ± 0.13	0.77 ± 0.09	1.74 ± 0.17
S7	1.21 ± 0.04	0.28 ± 0.05	0.28 ± 0.09	1.69 ± 0.02
S8	0.94 ± 0.03	1.04 ± 0.09	0.85 ± 0.03	1.51 ± 0.10
S9	0.86 ± 0.03	0.98 ± 0.15	0.48 ± 0.12	2.26 ± 0.15

**Table 3:** Assignment of ATR-FTIR identified peaks.

	Peak Wavenumber (cm^−1^)				
Sample	OH	AI	AII	AIII	C-O
S1	3033	1654	1541	1249	1026
S2	3299	1643	1552	1259	1041
S3	--	1654	1544	1259	1002
S4	3350	1672	1531	1257	1004
S5	3298	1637	1541	1265	1014
S6	3296	1650	1550	1257	1060
S7	3298	1637	1541	1249	1045
S8	3298	1647	1546	1259	1026
S9	--	--	--	1263	1017

OH: Hydroxyl group, AI: Amide I C=O stretching, AII: Amide II NH + CN bends, AIII: Amide III NH + CN stretches and C-O: Fingerprint C-O skeletal stretch.

**Table 4:** Coefficients of 4-D surface responses of the YM, MTS and RUS.

Coef.	YM	MTS	RUS
a	278,080	−87,244	532
A	316,167	376,667	−189
B	9467	25,333	0.3
C	−466,333	−489,833	−57
D	−1560	−5274	−20
A^2^	−28,111	−46,667	27
B^2^	-42.67	−218.7	−0.01
C^2^	51,333	65,667	7
D^2^	424.9	17.09	0.9

## References

[1] Kolarsick P.A., Kolarsick M.A., Goodwin C. (2011). Anatomy and physiology of the skin. J. Dermatol. Nurses Assoc..

[2] McBride A., Bargmann S., Pond D., Limbert G. (2016). Thermoelastic modelling of the skin at finite deformations. J. Therm. Biol..

[3] Oomens C., Van Vijven M., Peters G., Payan Y., Ohayon J. (2017). Skin Mechanics. Biomechanics of Living Organs: Hyperelastic Constitutive Laws for Finite Element Modeling.

[4] Weinstein G.D., Boucek R.J. (1960). Collagen and elastin of human dermis. J. Invest. Dermatol..

[5] McGrath J., Uitto J., Burns T. (2010). Anatomy and Organization of Human Skin. Rook’s Textbook of Dermatology.

[6] Silver F.H., Freeman J.W., DeVore D. (2001). Viscoelastic properties of human skin and processed dermis. Skin Res. Technol..

[7] Healthline. www.healthline.com/health/stratum-corneum.

[8] Mazzini M. (1968). Dermatología Práctica.

[9] Georges L., Querleux B. (2014). State-of-the-art constitutive models of skin biomechanics. Computational Biophysics of the Skin.

[10] Georges L., Georges L. (2019). Skin Biophysics: From Experimental Characterization to Advanced Modelling.

[11] Goldsmith L.A. (1990). My organ is bigger than your organ. Arch. Dermatol..

[12] Yuan J.H., Shi Y., Pharr M., Feng X., Rogers J.A., Huang Y.A. (2016). Mechanics Model for Sensors Imperfectly Bonded to the Skin for Determination of the Young’s Moduli of Epidermis and Dermis. J. Appl. Mech..

[13] Bischoff J.E., Arruda E.M., Grosh K.J. (2000). Finite element modeling of human skin using an isotropic, nonlinear elastic constitutive mode. J. Biomech..

[14] Dickey M.D. (2017). Stretchable and soft electronics using liquid metals. Adv. Mater..

[15] Yuan J., Dagdeviren C., Shi Y., Ma Y., Feng X., Rogers J.A., Huang Y. (2016). Computational models for the determination of depth-dependent mechanical properties of skin with a soft, flexible measurement device. Proc. Math. Phys. Eng. Sci..

[16] Dureja H., Tiwary A.K., Gupta S. (2001). Simulation of skin permeability in chitosan membranes. Int. J. Pharm..

[17] Dąbrowska A., Rotaru G.M., Spano F., Affolter C., Fortunato G., Lehmann S., Derler S., Spencer N.D., Rossi R.M. (2017). A water-responsive, gelatine-based human skin model. Tribol. Int..

[18] Baldino L., Cardea S., Scognamiglio M., Reverchon E. (2019). A new tool to produce alginate-based aerogels for medical applications, by supercritical gel drying. J. Supercrit. Fluids.

[19] Marchal C., Nadi M., Tosser A.J., Roussey C., Gaulard M.L. (1989). Dielectric properties of gelatine phantoms used for simulations of biological tissues between 10 and 50 MHz. Int. J. Hyperth..

[20] Navarro L., Ceaglio N., Rintoul I. (2017). Structure and properties of biocompatible poly (glycerol adipate) elastomers modified with ethylene glycol. Polym. J..

[21] Navarro L., Mogosanu D.E., Ceaglio N., Luna J., Dubruel P., Rintoul I. (2017). Novel poly (diol sebacate) s as additives to modify paclitaxel release from poly (lactic-co-glycolic acid) thin films. J. Pharm. Sci..

[22] Imeson A. (2010). Food Stabilisers, Thickeners and Gelling Agents.

[23] Król Ż., Malik M., Marycz K., Jarmoluk A. (2016). Physicochemical properties of biopolymer hydrogels treated by direct electric current. Polymers.

[24] Basha M.A. (2018). Optical properties and colorimetry of gelatine gels prepared in different saline solutions. J. Adv. Res..

[25] Gelatina Elástica. https://education.mrsec.wisc.edu/gelatina-elastica/.

[26] Díaz O., Ferreiro T., Rodríguez-Otero J.L., Cobos Á. (2019). Characterization of chickpea (*Cicer arietinum* L.) flour films: Effects of pH and plasticizer concentration. Int. J. Mol. Sci..

[27] Cai L., Shi H., Cao A., Jia J. (2019). Characterization of gelatin/chitosan polymer films integrated with docosahexaenoic acids fabricated by different methods. Sci. Rep..

[28] Huang T., Tu Z.C., Shangguan X., Sha X., Wang H., Zhang L., Bansal N. (2019). Fish gelatin modifications: A comprehensive review. Trends Food Sci. Technol..

[29] Suderman N., Isa M.I.N., Sarbon N.M. (2018). The effect of plasticizers on the functional properties of biodegradable gelatin-based film: A review. Food Biosci..

[30] Mao J., Zhao L., De Yao K., Shang Q., Yang G., Cao Y. (2003). Study of novel chitosan-gelatin artificial skin in vitro. J. Biomed. Mater. Res. A.

[31] Rodríguez-Rodríguez R., Espinosa-Andrews H., Velasquillo-Martínez C., García-Carvajal Z.Y. (2020). Composite hydrogels based on gelatin, chitosan and polyvinyl alcohol to biomedical applications: A review. Int. J. Polym. Mater..

[32] Owda A.Y., Casson A.J. (2020). Electrical properties, accuracy, and multi-day performance of gelatine phantoms for electrophysiology. BioRxiv.

[33] Velcescu A., Lindley A., Cursio C., Krachunov S., Beach C., Brown C.A., Jones A.K., Casson A.J. (2019). Flexible 3D-printed EEG electrodes. Sensors.

[34] Lir I., Haber M., Dodiuk-Kenig H. (2007). Skin surface model material as a substrate for adhesion-to-skin testing. J. Adhes. Sci. Technol..

[35] Keshavarzi F., Zajforoushan Moghaddam S., Barré Pedersen M., Østergaard Knudsen N., Jafarzadeh S., Thormann E. (2021). Water vapor permeation through topical films on a moisture-releasing skin Model. Ski. Res. Technol..

[36] da Silva F.R., Silva R.O., de Castro Oliveira H.M., Dourado L.F.N., da Costa B.L., Lima B.S., Nunes P.S. (2020). Gelatin-based membrane containing usnic acid-loaded liposomes: A new treatment strategy for corneal healing. Biomed. Pharmacother..

[37] Shirazaki P., Varshosaz J., Kharazi A.Z. (2017). Electrospun gelatin/poly (glycerol sebacate) membrane with controlled release of antibiotics for wound dressing. Adv. Biomed. Res..

[38] Schottner G. (2001). Hybrid sol—gel-derived polymers: Applications of multifunctional materials. Chem. Mater..

[39] Bellini F., Alberini I., Ferreyra M.G., Rintoul I. (2015). Absolute Determination of the Gelling Point of Gelatin under Quasi-thermodynamic Equilibrium. J. Food Sci..

[40] Dieter G.E., Schmidt L.C. (2011). Engineering Design.

[41] Alarcon-Segovia L.C., Daza-Agudelo J.I., Glisoni R.J., Acha C., De Zan M.M., Rintoul I. (2020). A multiparametric model for the industrialization of co-precipitation synthesis of nano-commodities. Nanotechnology.

[42] Daza-Agudelo J.I., Badano J.M., Rintoul I. (2018). Kinetics and thermodynamics of swelling and dissolution of PVA gels obtained by freeze-thaw technique. Chem. Phys..

[43] Recursos Educativos de Química Orgánica. http://www.ugr.es/~quiored/lab/tablas_espec/ir.htm.

[44] Tongnuanchan P., Benjakul S., Prodpran T. (2013). Physico-chemical properties, morphology and antioxidant activity of film from fish skin gelatin incorporated with root essential oils. J. Food Eng..

[45] Chao S.C., Wang M.J., Pai N.S., Yen S.K. (2015). Preparation and characterization of gelatin–hydroxyapatite composite microspheres for hard tissue repair. Mater. Sci. Eng. C.

[46] Rahman M., Brazel C.S. (2004). The plasticizer market: An assessment of traditional plasticizers and research trends to meet new challenges. Prog. Polym. Sci..

[47] Gontard N., Guilbert S., Cuq J.L. (1993). Water and glycerol as plasticizers affect mechanical and water vapor barrier properties of an edible wheat gluten film. J. Food Sci..

[48] Jacobsen S., Fritz H.G. (1999). Plasticizing polylactide the effect of different plasticizers on the mechanical properties. Polym. Eng. Sci..

[49] Jia P., Xia H., Tang K., Zhou Y. (2018). Plasticizers derived from biomass resources: A short review. Polymers.

[50] Vanin F.M., Sobral P.J.A., Menegalli F.C., Carvalho R.A., Habitante A.M.Q.B. (2005). Effects of plasticizers and their concentrations on thermal and functional properties of gelatin-based films. Food Hydrocoll..

[51] Cao N., Yang X., Fu Y. (2009). Effects of various plasticizers on mechanical and water vapor barrier properties of gelatin films. Food Hydrocoll..

[52] Hamzeh S., Motamedzadegan A., Shahidi S.A., Ahmadi M., Regenstein J.M. (2019). Effects of drying condition on physico-chemical properties of foam-mat dried shrimp powder. J. Aquat. Food Prod. Tech..

[53] Gómez-Estaca J., Gómez-Guillén M.C., Fernández-Martín F., Montero P. (2011). Effects of gelatin origin, bovine-hide and tuna-skin, on the properties of compound gelatin—chitosan films. Food Hydrocoll..

